# Kinetics and Solid Effect Investigations During Oil Droplet Desorption from Oil-Contaminated Soil Using the Chemical Cleaning Method

**DOI:** 10.3390/molecules30122502

**Published:** 2025-06-07

**Authors:** Song Jiang, Lu Wang, Shuo Wang, Jiling Liang, Guang Lu, Lin Li, Yan Zhang, Qinghua Wang, Lunqiu Zhang

**Affiliations:** 1 School of Civil Engineering, Liaoning Petrochemical University, Fushun 113001, China; 17640441082@163.com (S.J.); wanglu990203@163.com (L.W.); 18340364282@163.com (S.W.); luguang@lnpu.edu.cn (G.L.); zhangyan_286192@163.com (Y.Z.); wqh__hqw@163.com (Q.W.); zhangdoctor2022@126.com (L.Z.); 2 Jilin Provincial Key Laboratory of Emerging Contaminants Identification and Control, Jilin Normal University, Siping 136000, China; 18186830368@163.com

**Keywords:** deoiling of oil-contaminated soil, chemical cleaning method, solid effect, desorption of oil droplets from soil

## Abstract

Considering the implications for the environment and human health, oil-contaminated soil generated in the petroleum industry requires treatment. Chemical cleaning represents an effective treatment approach for oil-contaminated soil and has attracted considerable attention. In this study, sodium d-gluconate (C_6_H_11_NaO_7_), trisodium citrate (C_6_H_5_Na_3_O_7_), and L-arginine (C_6_H_14_N_4_O_2_) were employed as detergents to remove oil from oily sludge. The impacts of sludge (solid) concentration (*C*_S_), types of detergents, temperature (*T*), and pH value on the deoiling efficiency (*D*_e_) were systematically investigated. The results indicated that at a given detergent concentration (*C*_DG_) and *C*_S_, *D*_e_ followed the order C_6_H_11_NaO_7_ > C_6_H_5_Na_3_O_7_ > C_6_H_14_N_4_O_2_. When *C*_S_ was 3.86 g·L^−1^ and *C*_DG_ was 10.0 g·L^−1^, sodium d-gluconate achieved a maximum *D*_e_ of approximately 85%. Additionally, at a fixed *C*_S_, *D*_e_ decreased as the pH value increased, while it increased with increasing temperature. Interestingly, during the deoiling equilibrium, an obvious “solid effect” (or *C*_S_−effect) was observed. The “solid effect” refers to the phenomenon where the oil distribution coefficient (*K*_D_) changes with an increase in *C*_S_. The observed *C*_S_ effect was described using the surface component activity (SCA) model. The values of the intrinsic distribution coefficient ($K_{D}^{0}$) and *C*_S_−effect constant (*γ*), which are the model parameters of the SCA model, were derived from three detergent−sludge systems under different temperatures (*T*) and pH values. The strength of the *C*_S_ effect (or *γ* value) was found to be independent of detergent type and increased as *T* and pH value increased. This study broadens the application range of the SCA model and contributes to a deeper understanding of the adsorption and desorption behavior of oil droplets at the solid−liquid interface.

## 1. Introduction

Oil-contaminated soil is mainly generated during the production, refining, storage, and transportation of petroleum [1,2]. Usually, it comprises 30–85 wt% water, 15–50 wt% oil, and 5–46 wt% solids like soil or sand [1,3,4]. Due to its hazards to the ecological environment and human health, the treatment of oil-contaminated soil is of great concern [1,2,3,4,5]. Researchers have suggested a number of ways to deal with it, such as the chemical cleaning method [2,6,7,8,9], solvent extraction [10,11,12,13,14,15,16], centrifugation [1,7,17,18], flotation [19,20], pyrolysis [21,22,23], freeze/thawing [24,25,26,27,28,29], sonication [5,30,31], supercritical treatment [32,33], microwave radiation [34,35,36], bio-treatment technology [37], and a combination of processes [6,38,39,40,41]. In our previous studies, the chemical cleaning method and solvent extraction were used to remove oil from oily sludge [2,10]. For the former, the effects of the type of surfactant, such as sodium dodecyl benzene sulfonate (SDBS, anionic), cetyltrimethylammonium bromide (CTAB, cationic), Tween 60 (non-ionic), and the cheap alkaline solution of NaOH, on oil removal efficiency were investigated [2]. These chemical detergents were effective in removing oil from the surface of oil-contaminated soil at a relatively lower cost. However, oily wastewater generated in the deoiling process is more difficult to treat, restricting its application in oily wastewater treatment projects because of the presence of surfactants and alkaline conditions. For the latter, the oil removal efficiency was better than for the chemical cleaning method, while the cost was relatively higher [10].

As reported, the deoiling equilibrium was regarded as a distribution balance of oil between the liquid phase (detergent solution) and the solid phase (sludge) [2,10]. The ratio of the oil concentration in the liquid phase (*C*_o_) to that in the solid phase (sludge) (denoted as *Γ*_o_) at distribution equilibrium is usually denoted as a distribution coefficient (*K*_D_) [2,10,15]. Thermodynamically, the *K*_D_ in a given system should be constant, independent of the concentrations of oil and solid (sludge) under constant *T*, pressure, and medium composition (e.g., pH, ionic strength). However, an abnormal phenomenon of *K*_D_ increasing with increasing *C*_S_ was observed in our previous studies on the deoiling of oily sludge with the corresponding particle diameters of 322 nm and 340 nm, using chemical cleaning and solvent extraction methods [2,10]. Such a phenomenon is described as the “solid concentration effect” or the “solid effect” (abbreviated as *C*_S_−effect). The phenomenon of variations in *K*_D_ with increasing *C*_S_ suggests that the experimentally measured *K*_D_ is not a thermodynamic equilibrium parameter [2,10,42,43]. The discrepancy in *K*_D_ is attributed to the deviation between the real and the ideal systems, caused by the presence of interactions among solid particles in the real system, which are absent in an ideal system [2,10,42,43,44]. Uncertainties in *K*_D_ at varying *C*_S_ will hamper the technological design of treatment processes.

Several models have been proposed to explain the *C*_S_ effect in the adsorption−desorption equilibrium at solid−liquid interfaces, such as the particle interaction model [45], metastable-equilibrium adsorption (MEA) theory [46], flocculation model [47], power function (Freundlich-like) model [48], etc. However, these models have some limitations in their application. For example, the classical Freundlich equation is more suitable for ideal adsorption equilibrium, and some model parameters cannot be measured. Considering the deviations between real and ideal systems caused by solid particle−particle interaction, a surface component activity (SCA) model was proposed. In this model, the activity coefficients of solid surface sites were assumed to be functions of *C*_S_ [43,44]. The SCA model has been used to analyze the *C*_S_ effect observed in the solvent extraction and the chemical cleaning process of oily sludge [2,10]. A *C*_S_-dependent function of *K*_D_ was derived, namely, SCA distribution coefficient function or SCA-*K*_D_ function [2,10]. An intrinsic (or thermodynamic) distribution coefficient ($K_{D}^{0}$), which is independent of *C*_S_, was used to characterize the deoiling equilibrium and represents a stronger intrinsic deoiling ability. It was preliminarily demonstrated that the SCA-*K*_D_ function effectively describes the observed *C*_S_ effect in these deoiling equilibria [2,10]. Perhaps, $K_{D}^{0}$ can be used to guide the handling of uncertainty issues in *K*_D_ at varying *C*_S_ levels in technological design parameters. Therefore, obtaining the value of $K_{D}^{0}$ for different systems is very important.

However, there remain some unclear issues, such as whether the solid particle size has any impact on the *C*_S_ effect and applicability of the SCA model and whether the physical or chemical factors affect the desorption of oil droplets from the surface of oil-contaminated soil. Therefore, in this study, environmentally friendly and easily degradable detergents, including sodium d-gluconate (C_6_H_11_O_7_Na), L−arginine (C_6_H_14_N_4_O_2_), and trisodium citrate (C_6_H_5_O_7_Na_3_), were employed to remove oil from oil-contaminated soil. This approach is expected to reduce costs and lower the difficulty of treating the oily wastewater generated in the deoiling process. Meanwhile, this study can provide a theoretical basis for the design of the petroleum sludge treatment process. The effects of detergent concentration (*C*_DG_), solid concentration (*C*_S_), temperature (*T*), and pH on the deoiling efficiency (*D*_e_) and distribution coefficient (*K*_D_) were examined. The kinetics of oil droplet desorption from the surface of oil-contaminated soil was analyzed using pseudo-first-order and pseudo-second-order equations. The SCA-*K*_D_ function was used to describe the observed *C*_S_ effect in this deoiling equilibrium.

## 2. Results and Discussion

### 2.1. Kinetics of Oil Droplet Desorption from Oil-Contaminated Soil

The oil removal process is actually a redistribution of oil droplets between the surface of soil particles (solid phase) and the detergent solution (liquid phase). The behavior of oil droplets leaving the surface of oil-contaminated soil and moving into detergent solution is regarded as the desorption of oil droplets. At the same time, soil particles also re-attract oil droplets from the liquid, i.e., adsorption of oil droplets by soil particles. Therefore, the deoiling process is a desorption–adsorption process of oil droplets on the surface of soil particles. At the initial stage of the deoiling reaction (such as *t* < 120 min), desorption is the main process. When the reaction time is enough (such as *t* > 180 min), the desorption–adsorption of oil droplets will reach a dynamic equilibrium, i.e., deoiling equilibrium.

**Figure 1:**
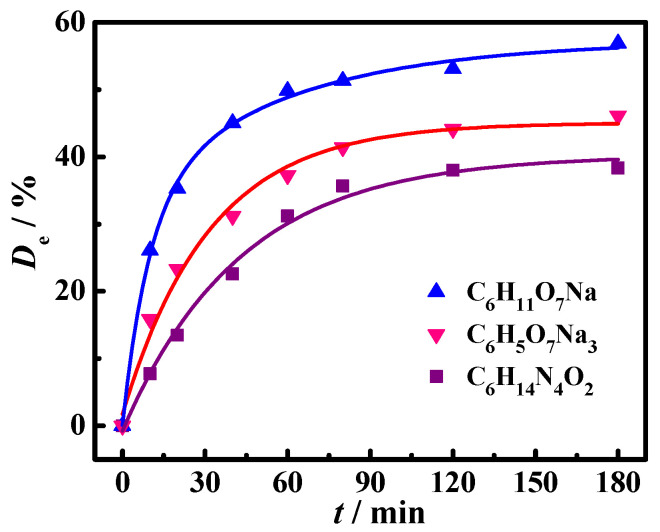
The relation curves of *D*_e_ and reaction time for three detergents at 25.0 °C when *C*_S_ was 30.78 g·L^−1^ and *C*_DG_ was 10.0 g·L^−1^.

Figure 1 shows the effect of reaction time on *D*_e_ for three detergents at 25.0 °C when *C*_S_ is 30.78 g·L^−1^ and *C*_DG_ is 10.0 g·L^−1^. *D*_e_ increased as reaching time increased and then tended to reach equilibrium value after 120 min. Therefore, the subsequent thermodynamic deoiling test time was set to 180 min to ensure that the deoiling reaction reached equilibrium.

The kinetics of the desorption of oil droplets from soil particles was investigated using pseudo-first-order and pseudo-second-order models, which were represented using Equations (1) and (2) [49]. For the desorption process of oil droplets from soil particles, the pseudo-second-order was represented using a modified form of Equation (2).

(1) $$\ln(\Gamma_{t}-\Gamma_{e})=\ln\Gamma_{e}-k_{1}t$$

(2) $$\frac{t}{\Gamma_{e}}=\frac{t}{\Gamma_{t}}+\frac{1}{k_{2}\Gamma_{e}^{2}},\;\mathrm{or}\;\frac{t}{\Gamma_{t}}=\frac{t}{\Gamma_{e}}-\frac{1}{k_{2}\Gamma_{e}^{2}}$$

Here, *Γ*_t_ (mg·g^−1^) is the oil content in the dry oil-contaminated soil at deoiling time (*t*, min), and *Γ*_e_ (mg·g^−1^) is the oil content in the dry oil-contaminated soil after reaching a deoiling equilibrium.

**Figure 2:**
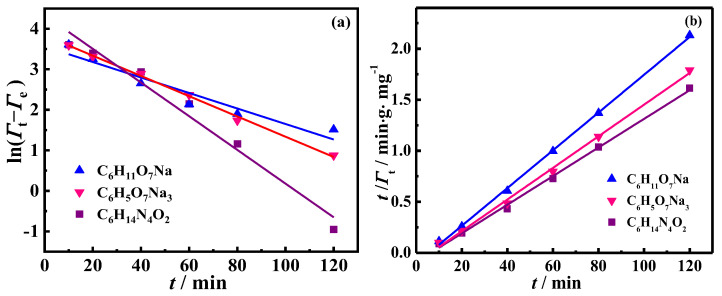
Linear regression of kinetic models: (**a**) pseudo-first-order and (**b**) pseudo-second-order.

The deoiling experimental data in the desorption process were fitted using Equations (1) and (2), as shown in Figure 2a,b. The fitting parameters obtained are listed in Table 1. For the values of *R*^2^, the pseudo-second-order model was the best model for fitting kinetic deoiling data, indicating that desorption of oil droplets was the rate-limiting step [49]. Moreover, the values of *Γ*_e_ obtained from the pseudo-second-order model were consistent with the experimental values of *Γ*_e, exp_. This consistency validates the applicability of the pseudo-second-order model for predicting the experimental kinetic data of the three liquid systems.

### 2.2. Deoiling from Oil-Contaminated Soil

#### 2.2.1. Effect of C_DG_ on D_e_

**Figure 3:**
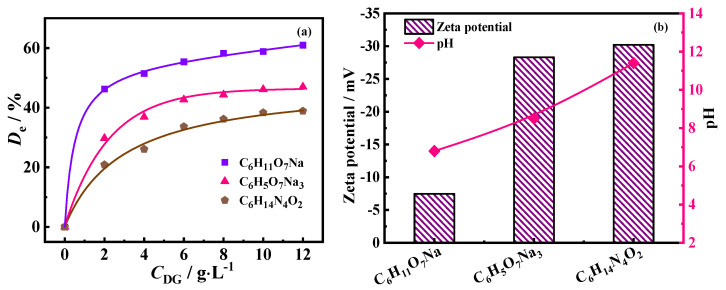
The relation curves of *D*_e_ and concentration (*C*_DG_) for three detergent solutions at *C*_S_ = 30.78 g·L^−1^ and *T* = 25.0 °C (**a**) and the zeta potential and pH values of three detergent aqueous solutions at *C*_DG_ = 10.0 g·L^−1^ and *T* = 25.0 °C (**b**).

As a critical factor, the detergent concentration (*C*_DG_) of three detergent aqueous solutions was investigated for oily sludge deoiling. The effect of *C*_DG_ on the deoiling efficiency (*D*_e_) was measured when *C*_S_ was 30.78 g·L^−1^ at 25.0 °C, as shown in Figure 3a. As can be seen, *D*_e_ values for the three detergents initially increased and then reached an equilibrium values with increasing *C*_DG_, which is consistent with previous findings [2]. The maximum *D*_e_, about 61%, was obtained using C_6_H_11_O_7_Na aqueous solution at *C*_DG_ = 12.0 g·L^−1^. Nevertheless, the three detergents had different deoiling capacities. *D*_e_ followed the order C_6_H_11_O_7_Na > C_6_H_5_O_7_Na_3_ > C_6_H_14_N_4_O_2_ at *C*_DG_ = 10.0 g·L^−1^ and *C*_S_ = 30.78 g·L^−1^.

To analyze the reason, the viscosity and the zeta potential of the three detergent aqueous solutions at *C*_DG_ = 10.0 g·L^−1^ were measured using an NDJ−5S Digital viscometer (Shanghai Yixin Scientific Instrument Co., Shanghai, China) and a Zetasizer Nano ZS90 Mastersizer (Malvern Instruments Co., Malvern, UK), respectively. The viscosity values of C_6_H_11_O_7_Na, C_6_H_5_O_7_Na_3_, and C_6_H_14_N_4_O_2_ aqueous solutions were 1.09 mPa·s, 0.96 mPa·s, and 0.92 mPa·s, respectively, decreasing in that order. Besides, the zeta-potential values of C_6_H_11_O_7_Na, C_6_H_5_O_7_Na_3_, and C_6_H_14_N_4_O_2_ solutions at *C*_DG_ = 10.0 g·L^−1^ and *T* = 25.0 °C were −7.47 mV, −28.30 mV, and −30.20 mV, respectively. The pH values of these solutions were 6.80, 8.50, and 11.38, respectively, as shown in Figure 3b. These factors may lead to a cumulative reduction in deoiling ability for C_6_H_11_O_7_Na, C_6_H_5_O_7_Na_3_, and C_6_H_14_N_4_O_2_ solutions.

#### 2.2.2. Effects of C_S_ and Temperature on D_e_

**Figure 4:**
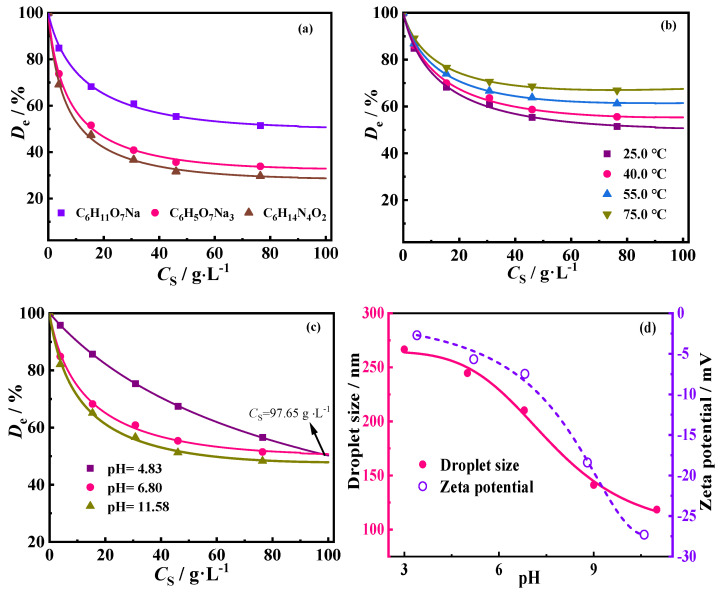
At *C*_DG_ = 10.0 g·L^−1^, plots of *D*_e_ versus *C*_S_ for different chemicals (**a**) and for C_6_H_11_O_7_Na solution under different temperatures at pH = 6.8 (**b**) and for C_6_H_11_O_7_Na solution under different pH values at *T* = 25.0 °C (**c**). Here, the dots indicate experimental data, and lines represent Equation (9) fits; zeta potential and droplet size of C_6_H_11_O_7_Na solution with different pH values at 25.0 °C (**d**).

The detergent aqueous solution with *C*_DG_ = 10.0 g·L^−1^ was used to remove oil from oil-contaminated soil to investigate the effect of *C*_S_ and temperature (*T*) on *D*_e_. Figure 4a shows the *D*_e_ of oily sludge for three detergent solutions at *C*_S_ values varying from 0 g·L^−1^ to 76.42 g·L^−1^ when *C*_DG_ was 10.0 g·L^−1^ at 25.0 °C. *D*_e_ initially decreased and then reached equilibrium as *C*_S_ increased. This result is similar to previous reports [2,10,14,15]. When *C*_S_ increased to 76.42 g·L^−1^, *D*_e_ approached an equilibrium value. The maximum equilibrium value of *D*_e_ was about 52% obtained by the C_6_H_11_O_7_Na solution with *C*_DG_ = 10.0 g·L^−1^ and *C*_S_ = 76.42 g·L^−1^. Additionally, the *D*_e_ sequence of the three detergents remained in the order C_6_H_11_O_7_Na solution > C_6_H_5_O_7_Na_3_ solution > C_6_H_14_N_4_O_2_ solution for a given *C*_S_.

Figure 4b shows the effect of temperature on *D*_e_ at various *C*_S_ values using the C_6_H_11_O_7_Na solution with *C*_DG_ = 10.0 g·L^−1^. The results show that *D*_e_ increased as *T* increased from 25.0 °C to 75.0 °C, which is in line with previous studies [2,10,50,51,52,53]. The suggested reason is that as *T* increases, the viscosity of the oil droplet at the solid–liquid interface decreases; molecular movements intensify, resulting in an increased probability and frequency of collisions between C_6_H_11_O_7_Na molecules in the liquid phase and solid particles; and more surface adsorption sites on the solid surface are occupied by H_2_O molecules. All these factors make it easier for oil droplet to escape from the solid surface and enter the liquid phase, resulting in an increase in *D*_e_ with increasing *T*.

#### 2.2.3. Effect of pH Value of Detergent Aqueous Solution on D_e_

**Figure 5:**
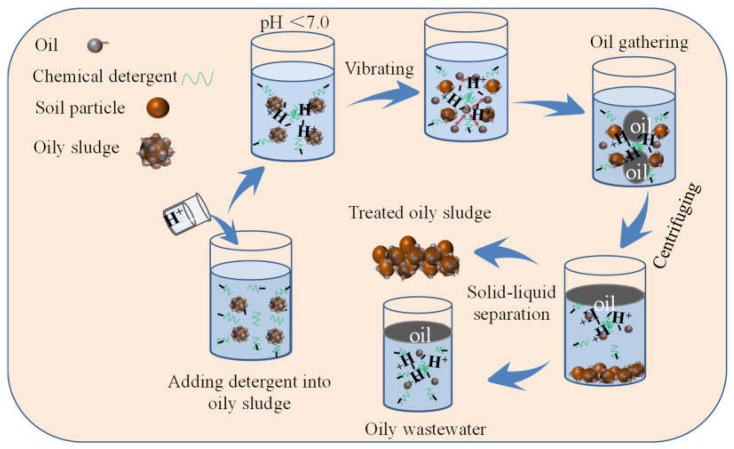
Schematic diagram of deoiling from oil-contaminated soil under acidic conditions at 25.0 °C.

The effect of the pH value of the C_6_H_11_O_7_Na solution with *C*_DG_ = 10.0 g·L^−1^ on *D*_e_ under different *C*_S_ at 25.0 °C was investigated. The results are shown in Figure 4c. It was found that *D*_e_ decreased with an increase in the pH value of the C_6_H_11_O_7_Na solution at a given *C*_S_, indicating that the acidic environment was more favorable for deoiling from oil-contaminated soil. To analyze the reason, the zeta potential and droplet size of the C_6_H_11_O_7_Na solution with different pH values were measured at *T* = 25.0 °C. The results are shown in Figure 4d. It was found that the zeta potential became more negative and the droplet size reduced with increasing pH values. This indicated that the C_6_H_11_O_7_Na solution became more stable when the pH increased. However, C_6_H_11_O_7_Na molecules were prone to aggregate and form large molecular clumps, and the surface was covered by H^+^ when pH < 7.0. Thus, C_6_H_11_O_7_Na molecular groups could adsorb the oil droplets, which would have a negative charge at the solid–liquid and oil–water interfaces, resulting in *D*_e_ being improved when pH < 7.0. The schematic diagram of deoiling from oil-contaminated soil under acidic conditions at 25.0 °C is shown in Figure 5.

It is assumed that the deoiling equilibrium system is a reallocation process of oil droplets in the solid–liquid phase. The experimentally measured distribution coefficient of oil (*K*_D_) could be obtained using Equation (5) as follows:

*K*_D_ = *C*_o_/*Γ*_o_ (3)

**Figure 6:**
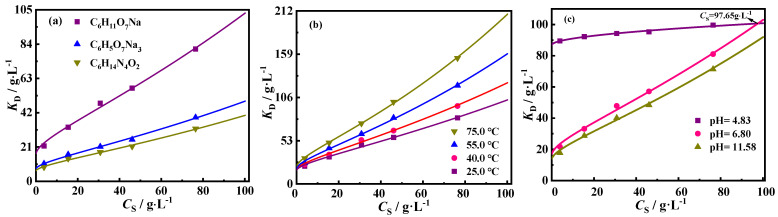
When *C*_DG_ was 10.0 g·L^−1^, plots of *K*_D_ versus *C*_S_ for three detergent solutions at 25.0 °C (**a**) and for C_6_H_11_O_7_Na solution under different temperatures (**b**) and under different pH values at 25.0 °C (**c**). Dots represent experimental data, and lines represent the SCA-*K*_D_ function fits.

Therefore, the relationship curves of calculated *K*_D_ values using Equation (3) and *C*_S_ in the three detergent–sludge systems at various *C*_S_ at 25.0 °C are shown in Figure 6a–c. As can be seen, *K*_D_ increased as *C*_S_ increased, which is consistent with reports in earlier studies [2,10,14,43,44]. This dependence of *K*_D_ on *C*_S_ indicates the presence of *C*_S_ effect in detergent–sludge deoiling systems. At a given *C*_S_ in the experimental range, *K*_D_ decreased sequentially for solutions of C_6_H_11_O_7_Na, C_6_H_5_O_7_Na_3_, and C_6_H_14_N_4_O_2_ when *C*_DG_ was 10.0 g·L^−1^ at 25.0 °C. Besides, *K*_D_ increased as *T* and pH values increased at a given *C*_S_. This indicates that *K*_D_ is not a thermodynamic equilibrium parameter [2,10], and the *K*_D_ value obtained at a specified *C*_S_ cannot explain the equilibrium behavior at other *C*_S_. This is because interactions among solid particles in a real dispersion system induce a deviation between real and ideal systems [2,10,42,43,44]. To account for this deviation, an SCA model was developed to explain the *C*_S_ effect [43,44] and can be used to assess the *C*_S_ effect in various deoiling equilibrium systems.

### 2.3. Deoiling Data Analysis Using the SCA Model

An SCA-*K*_D_ function was derived as follows to study the behavior of oil droplets at the solid–liquid interface [2,10]:

(4) $$K_{D}^{0}=f_{S}K_{D}$$

Here, $K_{D}^{0}$ is an intrinsic (or thermodynamic) distribution coefficient, and *f*_S_ is the activity coefficient of solid surface sites. For a given system under constant *T*, pressure, and medium composition, $K_{D}^{0}$ is independent of *C*_S_ and can be employed to characterize the deoiling equilibrium at any *C*_S_ [2,10,42].

In previous studies [2,10,43,44], an exponential form of the *C*_S_-dependent function of *f*_S_ was proposed as follows:

(5) $$f_{S}=\exp(-\gamma C_{S}^{0.5})$$

where *γ* is an empirical constant, called the *C*_S_-effect constant. The value of *γ* can assess the *C*_S_-effect strength; the higher the *γ* value, the stronger the *C*_S_ effect [2,10,42]. Then, the SCA-*K*_D_ function can be described as

(6) $$K_{D}=K_{D}^{0}\exp(\gamma C_{S}^{0.5})$$

or a linear form can be written as

(7) $$\ln K_{D}=\ln K_{D}^{0}+\gamma C_{S}^{0.5}$$

The function relationship of *D*_e_ and *K*_D_ was derived as [2,10]

(8) $$D_{e}=\frac{K_{D}}{K_{D}+C_{S}}$$

or

(9) $$D_{e}=\frac{K_{D}^{0}}{K_{D}^{0}+f_{S}\cdot C_{S}}$$

Equations (6)–(9) show that the experimentally measured *K*_D_ can estimate the values of $K_{D}^{0}$ and *γ*, which in turn can forecast the *C*_S_ dependence of *D*_e_.

**Figure 7:**
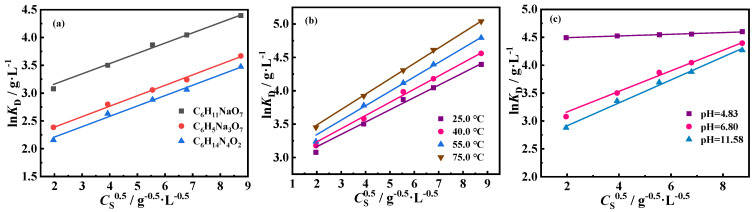
Plots of ln*K*_D_ versus $C_{S}^{0.5}$ for three detergents at 25.0 °C (**a**) and for C_6_H_11_O_7_Na solution under different temperatures (**b**) at pH = 6.8 and under different pH values at 25.0 °C (**c**) when *C*_DG_ was 10.0 g·L^−1^. Dots indicate experimental data, and lines indicate the SCA-*K*_D_ function fits.

In this chemical deoiling process, the SCA model (SCA-*K*_D_ function) was used to analyze the deoiling data, as well as investigate the effects of detergents, pH, and *T* on the *C*_S_ effect. For the three detergent–sludge systems at diverse *T* and pH values of detergent solutions, the ln*K*_D_ versus $C_{S}^{0.5}$ plots exhibited a linear relationship, as shown in Figure 7a−c. This observation is in accordance with the prediction of the SCA model, indicating that the SCA-*K*_D_ function is suitable for the chemical cleaning systems examined here. Table 2 shows the $K_{D}^{0}$ and *γ* values obtained from the intercepts and slopes of the ln*K*_D_−$C_{S}^{0.5}$ plots and the corresponding determination coefficient (*R*^2^) values. Thereafter, we used the values of the parameters of $K_{D}^{0}$ and *γ* (mentioned in Table 2) to simulate the changes in both *D*_e_ and *K*_D_ with *C*_S_ employing Equations (9) and (6), respectively. The results are shown in Figure 4a–c and Figure 6a–c, respectively. Each resulting simulated curve fitted well with the experimental data, achieving a higher *R*^2^ value, of more than 0.98. This indicates that the SCA model can be applied to explain the *C*_S_ effect accurately with acceptable parameters ($K_{D}^{0}$ and *γ*) values. Notably, the $K_{D}^{0}$ value is independent of *C*_S_; hence, $K_{D}^{0}$ can characterize the detergent’s intrinsic deoiling capacity; the higher the $K_{D}^{0}$ value, the better the intrinsic deoiling ability.

As mentioned in Table 2, the order of $K_{D}^{0}$ (or deoiling capacity) of the three detergents at 25.0 °C was C_6_H_11_O_7_Na > C_6_H_5_O_7_Na_3_ > C_6_H_14_N_4_O_2_, which was consistent with that obtained from the *D*_e_ data, as shown in Figure 4a.

For the C_6_H_11_O_7_Na aqueous solution−sludge system, when the temperature (*T*) increased from 25.0 °C to 75.0 °C, $K_{D}^{0}$ rose slightly from 16.34 g∙L^−1^ to 20.62 g∙L^−1^, resulting in a slight increase in *D*_e_. This is consistent with the results shown in Figure 4b. When the pH value increased from 4.83 to 11.58 at 25.0 °C, $K_{D}^{0}$ decreased from 86.80 g∙L^−1^ to 12.20 g∙L^−1^. Besides, the *γ* values increased with increasing *T* and pH, indicating that the changes in *K*_D_ with varying *T* and pH values were caused by the influence of *T* and pH on the *C*_S_ effect (or *γ* value). Similar results have been reported [2,10]. The cause of this phenomenon remains unknown to date. A possible reason is that the frequency of collisions among solid particles increases with an increase in *T*, while the inter-particle force is strengthened with an increase in pH, leading to a greater deviation between the real deoiling system and its ideal state. As a result, the *γ* value increased with increasing *T* and pH.

Furthermore, the *γ* values for the three chemicals were nearly similar at 25.0 °C, yielding an average *γ* value of 0.186 L^0.5^·g^−0.5^ with a ~1% maximum relative error, indicating that the strengths of the *C*_S_ effect were comparable among the three chemical–sludge systems at a constant *T*. Possible reasons are as follows: (1) the physicochemical properties of soil particles used in the deoiling process were hardly influenced by the three detergents, while the properties of the soil particles were the key factor influencing the *C*_S_ effect; (2) the adsorption sites on the solid surface were occupied by a large number of detergent molecules, causing the oil droplets to disperse into the liquid phase; (3) the three detergent solutions had similar viscosity, which was beneficial for the oil droplets, making them disperse into the liquid phase.

## 3. Experiments

### 3.1. Materials

**Figure 8:**
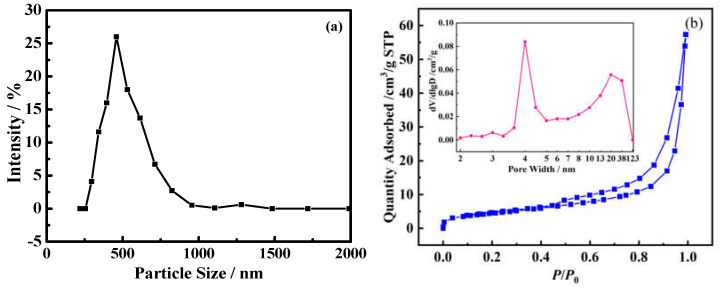
The particle size distribution (**a**) and BET curve of the soil (**b**).

Simulated oil-contaminated soil was prepared using oil from an oil field in Jilin province, China. It was mixed with some water and soil from the flower bed next to the Second Teaching Building of Liaoning Petrochemical University and sieved using a 100-mesh sieve, according to an oil–water–soil mass ratio of 1:1:8. To obtain homogenized oil-contaminated soil, the mixture of oil, water, and soil was mixed using a YD90S-8/4 cement mortar mixer (Wuxi Construction Engineering Test Equipment Co., Wuxi, China). The oil-contaminated soil obtained was measured using the mass-loss method, comprising 0.12 g·g^−1^ of oil (*Γ*_io_), 0.11 g·g^−1^ of water (*w*_w_), and 0.77 g·g^−1^ of solids (*w*_s_). Briefly, a mount of oil-contaminated soil (*m*_0_) was dried in an oven (Shanghai Jinghong Experimental Equipment Co., Shanghai, China) for 12 h at 105 °C, and the ratio of the loss mass to *m*_0_ is water content (*w*_w_). A mount of the obtained dry oil-contaminated soil (*m*_dry_) was then calcined at 600 °C for 3 h in a muffle furnace (Resistance Furnace Temperature Controller, Shaoxing Shangyu Road market branch analysis instrument factory, Shaoxing, China), and the loss mass is the organic in this process. Its content (*Γ*_io_) is the ratio of the organic mass to *m*_dry_. The particle size and BET specific surface area of the soil used were about 500 nm (fine-grained soil) measured using Zetasizer Nano S90 Mastersizer (Malvern Instruments Co., Malvern, UK) and 16.30 m^2^·g^−1^ measured using N_2_ adsorption−desorption (Quadrasorb SI−MP system, Quantachrome Instruments, Boynton Beach, FL, USA), as shown in Figure 8a,b. As Figure 8b shows, the maximum peak in the pore width distribution of the soil particles was 3.87 nm, and the total pore volume was 0.086 cm^3^/g.

The trisodium citrate (C_6_H_5_O_7_Na_3_·2H_2_O, AR) and L−arginine (L−C_6_H_14_N_4_O_2_, BR) were procured from Sinopharm Chemical Reagent Co. (Shanghai, China). Petroleum ether (30–60 °C, AR) and sodium d-gluconate (C_6_H_11_O_7_Na, AR) were purchased from Tianjin Kermel Chemical Reagent Co. (Tianjin, China). All chemicals were used as received. Deionized water used was obtained from a Hitech−Kflow water purification system (Hitech, Shenzhen, China).

### 3.2. Deoiling Tests

**Figure 9:**
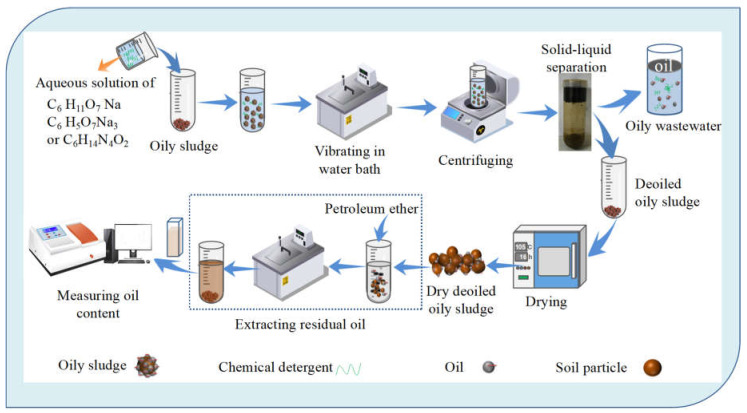
Schematic flow of the experiment.

Aqueous solutions of C_6_H_11_O_7_Na, C_6_H_14_N_4_O_2_, or C_6_H_5_O_7_Na_3_ with concentrations (*C*_DG_) of 10.0 g·L^−1^ were used as detergents to remove oil from oil-contaminated soil. Subsequently, a specific volume of the detergent aqueous solutions was added to a 50 mL centrifuge tube, and then a designated amount of oil-contaminated soil was added to it. As a result, the soil concentration (*C*_S_) in the detergent aqueous solution was 30.78 g·L^−1^, which could be calculated using Equation (10). The mixed suspension was shaken in a THZ−82 thermostatic water bath shaker (Wuhan Grey Mo Lai Detection Equipment Co., Wuhan, China) at 240 rpm for 0–180 min at 25.0 °C. Then, the mixture suspension was centrifuged using a TG18G tubular centrifuge (Changzhou Jintan Gaoco Instrument Factory, Changzhou, China) at a speed of 4000 rpm. The obtained precipitate was washed three times with water and dried at 105.0 °C for 16 h. The residual oil content (*Γ*_o_) in the obtained precipitate (solid) was determined using the petroleum ether extraction method reported in our previous papers [2,10]. Briefly, *Γ*_o_ was determined by monitoring the absorbance at λ = 227 nm, which was the absorption wavelength of oil in sludge, using a 745 PC uv–vis absorption spectroscope (Shanghai Yixin Scientific Instrument Co., China), and calculated by regression analysis according to the standard curve obtained from a series of standard petroleum ether solutions of the oil [10]. Based on the initial oil concentration (*Γ*_io_) and the sludge mass used, the deoiling efficiency (*D*_e_) and oil concentrations in the liquid phase (*C*_o_) were obtained using Equations (11) and (12). The schematic flow of the experiment is shown in Figure 9.

(10) $$C_{S}=\frac{m\cdot w_{s}}{\frac{m\cdot w_{w}}{\rho_{w}}\times 10^{-3}+V}$$

*D*_e_ = (*Γ*_io_–*Γ*_o_)/*Γ*_io_ (11)

*C*_o_ = (*Γ*_io_–*Γ*_o_) *m*/*V* (12)

Here, *Γ*_io_ and *Γ*_o_ are the initial and residual oil contents in the oily sludge (g·g^−1^), respectively; *m* is the mass of oil-contaminated soil (g); *ρ*_w_ is the density of water (g·cm^−3^); and *V* is the liquid phase volume (L).

Therefore, *C*_DG_ ranging from 0.0 g·L^−1^ to 12.0 g·L^−1^ and *C*_S_ varying in the range of 0–76.42 g·L^−1^ were used to investigate the influences of *C*_DG_, *C*_S_, and temperature (*T*, from 25.0 to 75.0 °C) on *D*_e_ using the same procedure, with a reaction time of 3.0 h.

Each test was performed in triplicate, and the final value was the average value.

## 4. Conclusions

Three chemicals, C_6_H_11_O_7_Na, C_6_H_5_O_7_Na_3_, and C_6_H_14_N_4_O_2_, were used as detergents to remove oil from oil-contaminated soil. The kinetic deoiling data were more in line with the pseudo-second-order model, with higher *R*^2^ values, indicating that the desorption of oil was a rate-controlling step. The pseudo-second-order model predicted the experimental kinetic data for the three liquid systems. The deoiling capacity of C_6_H_11_O_7_Na, C_6_H_5_O_7_Na_3_, and C_6_H_14_N_4_O_2_ followed a decreasing order at 25.0 °C, and the deoiling efficiency (*D*_e_) of the C_6_H_11_O_7_Na solution with *C*_DG_ = 10.0 g∙L^−1^ increased with increasing *T* and decreased with increasing pH. Moreover, an obvious *C*_S_-effect phenomenon was observed in the deoiling equilibrium, which could be accurately described using the SCA model (or SCA-*K*_D_ function). The strength of the *C*_S_ effect (or *γ* value) was independent of the detergents used in this study but increased slightly with increasing *T* and pH values. The variations in *K*_D_ with *T* and pH values for sodium d-gluconate were due to the influence of *T* and pH values on the *C*_S_ effect. Overall, this study enhanced the understanding of the deoiling behavior of oil-contaminated soil from a new perspective and confirmed that the SCA model could be applied as a mathematical tool to analyze the deoiling data encompassing the *C*_S_ effect.

## Figures and Tables

**Table 1:** Kinetics models parameters simulated using Equations (1) and (2) at *C*_DG_ = 10.0 g·L^−1^.

Liquid System	Pseudo-First-Order			Pseudo-Second-Order			*Γ*_e, exp_ (mg·g^−1^)
	*k*_1_ (min^−1^)	*Γ*_e, cal_ (mg·g^−1^)	*R* ^2^	*K*_2_ (g·mg^−1^·min^−1^)	*Γ*_e_ (mg·g^−1^)	*R* ^2^	
C_6_H_14_N_4_O_2_	0.042	76.40	0.967	0.00219	71.28	0.997	74.01
C_6_H_5_O_7_Na_3_	0.025	46.33	0.996	0.00244	64.52	0.997	64.63
C_6_H_11_O_7_Na	0.019	35.24	0.909	0.00324	54.02	0.999	51.77

**Table 2:** SCA model parameters simulated using Equation (6) at *C*_DG_ = 10.0 g·L^−1^.

Detergent	*T* (°C)	pH	$K_{D}^{0}$ (g·L^−1^)	*γ* (L^0.5^·g^−0.5^)	*R* ^2^
C_6_H_11_O_7_Na	25.0	4.83	86.80	0.015	0.982
	25.0	6.80	16.34	0.184	0.995
	25.0	11.58	13.30	0.193	0.997
	40.0	6.80	17.10	0.198	0.997
	55.0	6.80	18.38	0.216	0.998
	75.0	6.80	20.62	0.231	0.999
C_6_H_5_O_7_Na_3_	25.0	8.50	7.48	0.188	0.995
C_6_H_14_N_4_O_2_	25.0	11.38	6.28	0.186	0.994

## Data Availability

The authors confirm that the data supporting the findings of this study are available within the article.
